# The effects of molar activity on [^18^F]FDOPA uptake in patients with neuroendocrine tumors

**DOI:** 10.1186/s13550-021-00829-z

**Published:** 2021-09-08

**Authors:** Gilles N. Stormezand, Romano S. B. H. Schreuder, Adrienne H. Brouwers, Riemer H. J. A. Slart, Philip H. Elsinga, Annemiek M. E. Walenkamp, R. A. J. O. Dierckx, Andor W. J. M. Glaudemans, Gert Luurtsema

**Affiliations:** 1 Department of Nuclear Medicine and Molecular Imaging, University Medical Center Groningen, Medical Imaging Center, University of Groningen, Hanzeplein 1, 9700 RB Groningen, The Netherlands; 2 Department of Medical Oncology, University Medical Center Groningen, University of Groningen, Groningen, The Netherlands

**Keywords:** [^18^F]-FDOPA, Neuroendocrine tumors, Molar activity, Biodistribution

## Abstract

**Background:**

6-[^18^F]fluoro-l-3,4-dihydroxyphenyl alanine ([^18^F]FDOPA) is a commonly used PET tracer for the detection and staging of neuroendocrine tumors. In neuroendocrine tumors, [^18^F]FDOPA is decarboxylated to [^18^F]dopamine via the enzyme amino acid decarboxylase (AADC), leading to increased uptake when there is increased AADC activity. Recently, in our hospital, a new GMP compliant multi-dose production of [^18^F]FDOPA has been developed, [^18^F]FDOPA-H, resulting in a higher activity yield, improved molar activity and a lower administered mass than the conventional method ([^18^F]FDOPA-L).

**Aims:**

This study aimed to investigate whether the difference in molar activity affects the [^18^F]FDOPA uptake at physiological sites and in tumor lesions, in patients with NET. It was anticipated that the specific uptake of [^18^F]FDOPA-H would be equal to or higher than [^18^F]FDOPA-L.

**Methods:**

We retrospectively analyzed 49 patients with pathologically confirmed NETs and stable disease who underwent PET scanning using both [^18^F]FDOPA-H and [^18^F]FDOPA-L within a time span of 5 years. A total of 98 [^18^F]FDOPA scans (49 [^18^F]FDOPA-L and 49 [^18^F]FDOPA-H with average molar activities of 8 and 107 GBq/mmol) were analyzed. The SUVmean was calculated for physiological organ uptake and SUVmax for tumor lesions in both groups for comparison, and separately in subjects with low tumor load (1–2 lesions) and higher tumor load (3–10 lesions).

**Results:**

Comparable or slightly higher uptake was demonstrated in various physiological uptake sites in subjects scanned with [^18^F]FDOPA-H compared to [^18^F]FDOPA-L, with large overlap being present in the interquartile ranges. Tumor uptake was slightly higher in the [^18^F]FDOPA-H group with 3–10 lesion (SUVmax 6.83 vs. 5.19, *p* < 0.001). In the other groups, no significant differences were seen between H and L.

**Conclusion:**

[18F]FDOPA-H provides a higher activity yield, offering the possibility to scan more patients with one single production. Minor differences were observed in SUV’s, with slight increases in uptake of [^18^F]FDOPA-H in comparison to [^18^F]FDOPA-L. This finding is not a concern for clinical practice, but could be of importance when quantifying follow-up scans while introducing new production methods with a higher molar activity of [^18^F]FDOPA.

## Introduction

Neuroendocrine tumors (NETs) are a collection of rare tumors that are named based on the origin of their occurrence in the body and their histological features. They arise from neuro-endocrine tumor cells and may occur in various sites within the body. Patients with NETs may exhibit symptoms directly related to the release of endocrine substances from the tumor (secretory NETs) [1]. In these cases, even very small tumors (e.g., insulinomas) can cause symptoms. In other instances (in non-secretory NETs), symptoms may arise late in the disease, for example, due to local mass effect. NETs may also be detected incidentally, during imaging performed for other conditions, or as an unexpected pathologic finding after surgery.

Patient management depends on the extent and localization of the disease, which can be visualized using imaging techniques. Currently, imaging of NETs is usually based on a combination of anatomic and functional imaging. The PET tracer6-[^18^F]fluoro-l-3,4-dihydroxyphenyl alanine ([^18^F]FDOPA) is a commonly used functional imaging technique for the detection and staging of NETs [2–4]. Increased [^18^F]FDOPA uptake in NETS is associated with an active catecholamine pathway, resulting in higher uptake of precursors by the large amino acid transporter (LAT) and increased activity of the enzyme amino acid decarboxylase (AADC) [2]. In the cell, [^18^F]FDOPA is decarboxylated to [^18^F]dopamine via the AADC enzyme and can be further metabolized to noradrenalin and adrenalin. After excretion from the cell, these catecholamines can be taken up by selective transporter systems [2, 3]. Until recently in our hospital, the tracer [^18^F]FDOPA was produced using an electrophilic ^18^F-fluorination with trimethylstannyl precursor in combination with production of [^18^F]F_2_ gas performed by the bombardment of ^20^Ne using deuterons. This results in a low activity yield and leads to [^18^F]FDOPA with a low molar activity. For that reason, we developed a new GMP compliant multi-dose production of [^18^F]FDOPA using the double shoot proton method. This new radionuclide production method leads to a higher activity yield and improved molar activity resulting in a lower mass [5]. In daily clinical practice, this could allow more patients to be scanned with a single production.

Currently, it is not completely clear how differences in molar activity affect the human biodistribution of the radiopharmaceutical in vivo, both in organs and at tumor sites. Knowledge of the effect of molar activity on [^18^F]FDOPA uptake is relevant when evaluating patients with NET that have been scanned longitudinally using both [^18^F]FDOPA production techniques, particularly when performing quantitative comparisons to differentiate between partial response, stable or progressive disease. This study aimed to investigate whether the difference in molar activity affects the [^18^F]FDOPA uptake at physiological sites and in tumor lesions in patients with NET. It was anticipated that the specific uptake of [^18^F]-FDOPA-H would be at least equal to or higher than [^18^F]-FDOPA-L because of reduced competition with the non-radioactive form of the tracer at the binding site.

## Methods

### Patients

For this retrospective, single-center study, patients with pathologically confirmed NET who underwent [^18^F]FDOPA PET scanning at the department of Nuclear Medicine and Molecular in the UMCG as part of their routine follow-up were considered. The scans were performed within a period of 5 years (4 years prior and up to one year after the transition date of the radiopharmaceutical production method). If more than two scans of the same patients were available, the two scans closest to the transition date (one with the old and one with the new production method) were selected for this analysis. Patients’ clinical records were checked for current medication use and surgical intervention. Patients were allowed to have had minor surgery (resection of the gallbladder or a unilateral adrenal gland resection) in between scans. Patients who received major surgery (tumor resections from the gastrointestinal tract or resection of one of the kidneys) between scans were excluded from further analysis. Also excluded from the analyses were patients who showed progressive disease between the scans, according to RECIST criteria on the diagnostic CT, showed more than 5 new lesions on the second PET scan, or showed more than 10 tumor lesions in total on the baseline PET scan. To control the potential influence of the course of the disease on the uptake of [^18^F]FDOPA, all remaining subjects were subdivided into three groups. The first group consisted of patients without visible lesions on both [^18^F]FDOPA PET/CT scans (no metabolically active disease; follow-up scans after an earlier detected and resected NET or subjects at risk for NET without any found) to allow for measurements of background organ uptake. The second group were patients with no more than 2 lesions visible on both scans and with stable disease, whereas the third group consisted of the patients with 3–10 lesions on both scans, with no more than 5 new lesions on the second PET and who did not fulfil the criteria of PD according to RECIST. In the latter groups, both uptake in background and tumor tissue was evaluated. This approach was chosen to minimize the influence of disease progression on the uptake of the radiopharmaceutical.

### [^18^F]FDOPA production

Both [^18^F]FDOPA-L and H were produced via electrophilic fluorination of the trimethylstannyl precursor with [^18^F]F_2_. However, the used radionuclide method to produce [^18^F]F_2_ gas was different. [^18^F]FDOPA-H was produced from [^18^O]O_2_ via a double-shoot approach, according to a previously published protocol [5]. This new GMP compliant multi-dose production of [^18^F]FDOPA-H resulted in a higher activity yield and improved molar activity, resulting in a lower administered mass compared to [^18^F]FDOPA-L (Table 2). The conventional [^18^F]FDOPA-L was synthesized using [^18^F]F_2_ produced from ^20^Ne using deuterons [6]. This radionuclide production proves lower activity yield and lower molar activity, because more cold F_2_ gas is needed for optimal recovery of [^18^F]F_2_ gas from the target. The average activity yield of [^18^F]FDOPA-H was 4 ± 0.4 GBq with a molar activity of 107 (40–163) GBq/mmol. For [^18^F]FDOPA-L, the average activity yield was 0.5 ± 0.1 GBq with a molar activity of 8.23 (4–61) GBq/mmol.

### PET imaging and analysis procedure

All [^18^F]FDOPA PET/CT scans were performed from the top of the skull through mid-thigh 60 ± 6 min after intravenous administration of a standard dose of approximately 200 MBq [^18^F]FDOPA on a Biograph mCT camera (Siemens Medical Systems, Knoxville, TN, USA). All patients fasted for 6 h and were allowed to continue medication. All subjects were pretreated with 2.0 mg/kg of carbidopa orally with a maximum of 150 mg. The acquisition was performed in seven bed positions of 2 min emission time for patients between 60 and 90 kg. Patients with a bodyweight less than 60 kg and more than 90 kg body weight were scanned with 1 min and 3 min per bed position, respectively. Raw data were reconstructed according to guideline-based standardized NEDPAS or EARL algorithms for SUV calculations [7]. The uptake of [^18^F]FDOPA in target and background lesions was measured using manually drawn spherical volumes of interests (VOIs) on EARL reconstructed PET images, using Siemens Syngo.via software version VB10. Tumor lesions were defined as visual uptake higher than the background (bloodpool) and not due to a known physiological uptake pattern of [^18^F]FDOPA. [^18^F]FDOPA uptake in tumor lesions was expressed as maximum SUV (SUV_max_). SUV_mean_ values were obtained for the following background organs: striatum, heart, thoracic aorta, liver, gallbladder, pancreas (divided in head, body and tail because of considerable physiological variation in these regions), kidneys, and adrenal glands. Scans were divided into two categories based on the use of a low molar activity ligand ([^18^F]FDOPA-L) or a high molar activity ligand ([^18^F]FDOPA-H).

### Statistics

Group comparisons between [^18^F]FDOPA-L and [^18^F]FDOPA-H in background regions and tumor lesions were performed using Wilcoxon Signed-Rank tests (*p* < 0.05 considered significant for tumors, *p* < 0.004 in organs after correcting for multiple comparisons (0.05/12)). When comparing tumors, the same tumor was measured at baseline and follow-up. The effect size r was calculated for each comparison using Rosenthal’s formula. In the Wilcoxon Signed-Rank test, negative ranks indicate an increase in SUV from [^18^F]DOPA-L to [^18^F]FDOPA-H, whereas positive ranks indicate a decrease from [^18^F]FDOPA-H to [^18^F]FDOPA-L. Independent sample t tests were used for group comparisons in injected activity and mass, with *p* < 0.05 considered significant. For statistical analysis, SPSS was used (IBM Corp. Released 2015. IBM SPSS Statistics for Windows, Version 23.0. Armonk, NY: IBM Corp).

## Results

A total of 82 patients with 164 [^18^F]FDOPA PET scans were identified within the designated period before and after the transition date. After applying the exclusion criteria as previously described, 49 patients with 98 [^18^F]FDOPA scans remained for further analysis.

**Fig. 1:**
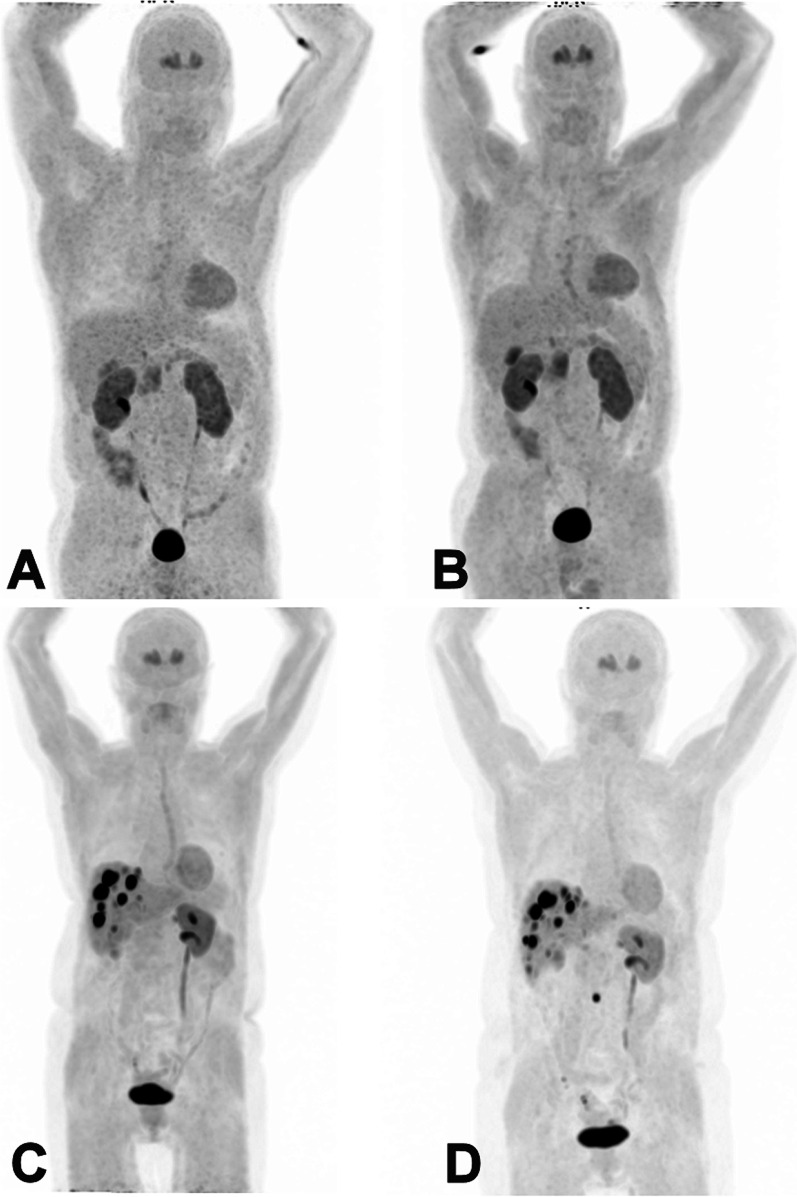
Representative maximum intensity projection (MIP) [^18^F]FDOPA-L PET images of the same subject without evidence of disease (group 1) scanned after administration of [^18^F]FDOPA-L (A) and [^18^F]FDOPA-H (B). Below is an example of a subject in group 3 with liver metastases scanned with [^18^F]FDOPA-L (C) and [^18^F]FDOPA-H (D). Only existing liver lesions were used for evaluation. A small new site of focal uptake was noted in a mesentery lymph node 20 months after the initial scan

Groups 1, 2 and 3 consisted of 22, 10 and 18 patients, respectively. Table 1 shows the group characteristics. Subjects had a mean age of 55.9 (group 1), 53.7 (group 2) and 63.5 (group 3), respectively. On average, the interval between scans was 2.4 years in group 1, 3.0 years in group 2 and 2.7 years in group 3. All patients did not receive any drug treatment related to the disease besides somatostatin analogues or medication targeted at symptoms. In group 1, one patient discontinued treatment after the first scan, in group 2 one patient started treatment after the first scan, and in group 3, eight patients were on treatment for both scans; however one patient stopped, whereas another initiated treatment after the first scan. No patient received any other form of systemic therapy in between the two scans. A total of 65 tumor lesions were evaluated twice, 9 in group 2 and 56 in group 3. The total number of lesions was stable in group 2. In group 3, the total number of lesions increased from 78 to 90, with 10 patients having an unchanged number of lesions, 3 patients having 1 new lesion, 3 patients with 2 new lesions, and 1 patient with 3 new lesions. Table 2 shows the radiopharmaceutical specifications. The injected mass was significantly higher using [^18^F]FDOPA-L than [^18^F]FDOPA-H in all groups. Figure 1 shows representative images of patients scanned with both [^18^F]FDOPA-L and [^18^F]FDOPA-H.

**Table 1:** Patient characteristics. Data are displayed in means (standard error of the mean)

	Group 1 (*n* = 22)	Group 2 (*n* = 9)	Group 3 (*n* = 18)
Age (years)	1st scan: 56 (4)	1st scan: 54 (4)	1st scan: 64 (3)
	2nd scan: 58 (4)	2nd scan: 57 (4)	2nd scan: 65 (3)
Sex (male, %)	53%	50%	41%
On somatostatin analogue treatment:			
At first scan (%)	4.5%	0%	44%
At second scan (%)	0%	11%	44%
Change of medication (%)	4.5%	11%	11% (1 stopped, 1 started therapy)
Total number of lesions evaluated	1st scan: 0	1st scan: 9	1st scan: 56
	2nd scan: 0	2nd scan: 9	2nd scan: 56
Total number of lesions	1st scan: 0	1st scan: 10	1st scan: 78
	2nd scan: 0	2nd scan: 10	2nd scan: 90
Interval surgery (*n*)	1 (cholecystectomy)	0	0
NET primary site (%)	GI-tract 68%	GI-tract 44%	GI-tract 65%
	Pancreas 5%	Thyroid 22%	Pancreas 6%
	Thyroid 5%	Lung 11%	Head and neck 6%
	Lung 5%	Adrenal 11%	Lung 12%
	Unknown 16%	Paraganglioma 11%	Adrenal 6%
			Ovary 6%
NET grade (%)	Grade 1 84%	Grade 1 33%	Grade 1 71%
	Undefined 16%	Grade 2 22%	Grade 2 12%
		Undefined 44%	Undefined 18%

**Table 2:** Radiopharmaceutical specifications. Data are displayed in means (standard error of the mean)

	Group 1 (no evidence of disease)	Group 2 (1–2 lesions)	Group 3 (3–10 lesions)
Activity administered (MBq)	[^18^F]FDOPA-L: 192 (2.3)	[^18^F]FDOPA-L: 205 (3.3)	[^18^F]FDOPA-L: 180 (7.9)
	[^18^F]FDOPA-H: 204 (4.0)	[^18^F]FDOPA-H: 192 (11.2)	[^18^F]FDOPA-H: 205 (2.1)
*p* value (*t* test)	0.05	0.10	< 0.001*
Injected mass (µg)	[^18^F]FDOPA-L: 5204 (356)	[^18^F]FDOPA-L: 4614 (1028)	[^18^F]FDOPA-L: 5167 (694)
	[^18^F]FDOPA-H: 515 (50)	[^18^F]FDOPA-H: 1205 (371)	[^18^F]FDOPA-H: 885 (283)
*p* value (*t* test)	< 0.001*	0.005*	< 0.001*

*Considered significant

**Fig. 2:**
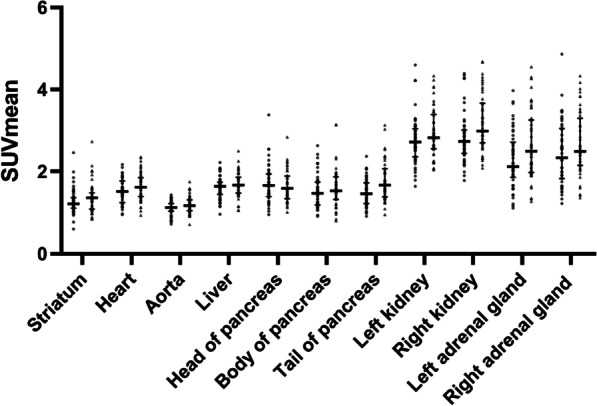
SUVmeans in background organs scanned with [^18^F]FDOPA-L and [^18^F]FDOPA-H. Bars represent medians and interquartile ranges. For each organ, the SUVmeans of [^18^F]FDOPA-L are displayed on the left (circles), and the SUVmeans of [^18^F]FDOPA-H are displayed on the right (triangles). Because of the large variation in SUVmean, the gallbladder was not plotted in this graph. This was probably related to variation in excretion

In Fig. 2, individual SUVmean values in background organs are plotted. In Table 3, the mean SUVmean values of the background organs are reported and the Wilcoxon-rank test results. No significant changes were observed in most organs after the implementation of [^18^F]FDOPA-H, with considerable overlap being present in the interquartile ranges. After correction for multiple comparisons, the only significant findings that remained were increased uptake in the left adrenal gland and right kidney in the [^18^F]FDOPA-H group compared to the [^18^F]FDOPA-L group (Fig. 2, Table 3).

**Table 3:** SUVmeans in background organs scanned with FDOPA-L and FDOPA-H in organs. Data are presented in medians plus interquartile ranges

Background region	SUVmean [^18^]FFDOPA-L	SUVmean [^18^]FFDOPA-H	*p* value	Effect size r
Striatum	1.21 (1.11–1.36)	1.36 (1.09–1.49)	0.011	− 0.36^a^
Heart	1.52 (1.25–1.77)	1.62 (1.39–1.85)	0.006	− 0.39^a^
Aorta	1.12 (1.04–1.22)	1.18 (1.05–1.31)	0.004	− 0.40^a^
Liver	1.64 (1.45–1.74)	1.67 (1.48–1.86)	0.068	− 0.26^a^
Gallbladder	2.65 (1–50-4.31)	2.90(0.96–4.17)	0.540	− 0.09^b^
Pancreas (head)	1.66 (1.39–1.94)	1.59 (1.34–1.89)	0.255	− 0.17^b^
Pancreas (body)	1.47 (1.19–1.75)	1.53 (1.32–1.87)	0.174	− 0.20^a^
Pancreas (tail)	1.46 (1.22–1.73)	1.55 (1.34–1.84)	0.010	− 0.38^a^
Left kidney	2.73 (2.36–3.04)	2.83 (2.55–3.39)	0.028	− 0.31^a^
Right kidney	2.74 (2.45–3.02)	2.99 (2.70–3.67)	0.000*	− 0.59^a^
Left adrenal gland	2.12 (1.86–2.72)	2.50 (1.98–3.26)	0.000*	− 0.52^a^
Right adrenal gland	2.34 (1.83–3.05)	2.49 (2.15–3.30)	0.005	− 0.41^a^

*Considered significant

^a^Based on negative ranks

^b^Based on positive ranks

Concerning tumor lesions, no significant differences were detected in group 2, whereas in group 3, SUVmax was significantly higher in the [^18^F]FDOPA-H group, although the medians and interquartile ranges were largely overlapping (Table 4).

**Table 4:** SUVmax in tumor lesions scanned with FDOPA-L and FDOPA-H. Data are presented in medians plus interquartile ranges

Group	SUVmax FDOPA-L	SUVmax FDOPA-H	*p* value	Effect size r
II (*n* = 9)	5.53 [4.29–8.62]	7.02 [3.14–10.79]	0.67	− 0.13^a^
III (*n* = 56)	5.19 [3.56–10.00]	6.83 [4.30–14.64]	< 0.001*	− 0.58^a^

*Considered significant

^a^Based on negative ranks

^b^Based on positive ranks

## Discussion

In this study, [^18^F]FDOPA-H was associated with comparable or slightly higher uptake in various background organs and neuro-endocrine tumor lesions, a significantly lower injected mass (order of magnitude 4 to tenfold lower) and higher molar activity. Higher uptake was observed in the left adrenal gland and the right kidney, but no significant differences were observed in other regions after correcting for multiple comparisons. Despite these differences, largely overlapping median SUV values and interquartile ranges suggest only moderate effects. This is in line with visual assessments being performed by nuclear medicine physicians, who did not observe significant alterations in tracer distribution related to the introduction of [^18^F]FDOPA-H. Kuik et al. reported no significant differences in tumor and physiological organ uptake when comparing SUVs obtained using both [^18^F]FDOPA with high molar activity and [^18^F]FDOPA with low molar activity in animal models, despite an order of magnitude of 3 times higher molar activity in the [^18^F]FDOPA-H group [8]. However, this analysis was performed using a small sample size, consisting of only 4 mice per group. Another prospective study compared high and low specific activity of [^123^]I-MIBG in human subjects with pheochromocytoma (*n* = 5), and also failed to find significant differences using both preparations [9].

In contrast, Akamatsu et al. reported significant increases in the striatal-to-occipital ratio (SOR) after introducing high molar activity [^18^F]FDOPA. However, the observed differences were most likely related to higher resolution PET camera systems used in their sample [10]. Indeed, partial volume effects associated with a lower scanning resolution may lead to underestimation of SORs [11]. The significantly increased [^18^F]FDOPA-H uptake in this study is most likely the result of reduced competition for binding sites with the non-radioactive form of the tracer. This may particularly be the case at sites with relatively high uptake, such as in the adrenals and tumors. The higher uptake in the right kidney may have a relationship with the estimated glomerular filtration rate, but this information was not always available at the time of scan.

Based on our findings, it cannot be ruled out that changes in molar activity may introduce small differences in SUVs, especially in the adrenal glands, kidneys and tumor lesions. Although this may not affect the assessment of the images in the daily clinic, which mainly relies on visual inspection, it may be of relevance in longitudinal research studies evaluating therapy-related changes in SUVs. Also, it could be important when using (semi)-quantitative thresholds, e.g., for identifying a pheochromocytoma [12] and to striatal-to-occipital ratios in patients evaluated for the presence of a presynaptic dopaminergic deficit [13]. In our study, however, no significant differences in the striatum were detected.

## Limitations

A significant limitation of our study is the retrospective design. This has led to variable periods between scans, which may be particularly relevant in the presence of disease. Because of the time interval, the reported increases in SUVmax in tumors could be related to disease progression and therefore this finding has to be interpreted with caution. We, however, aimed to minimize this issue by selecting those patients who had stable disease or only minimal progression on imaging. A number of patients received treatment with somatostatin analogues. Typically these agents have little, if any, effect on size and induce a significant growth delay improving the progression-free survival [14], [^18^F]-FDOPA uptake is not known directly to be affected by somatostatin analogue treatment. Therefore, we do not expect that our results have been significantly influenced by medication. Another limitation is that a heterogeneous group of NETs has been included, which could have hampered the detection of more specific tumor-related alterations in tracer distribution, such as those associated with monoamine oxidase (MAO)-A expression (mainly upregulated in pancreas and GE NETs [15]) or the vesicular monoamine transporter (VMAT, inversely correlated with prognosis [16]). However, prior research indicated that these factors only play a role under specific experimental conditions and this is unlikely to affect the in vivo biodistribution differences between [^18^F]FDOPA-H and [^18^F]FDOPA-L. Finally, relatively small sample sizes were used, which may have led to small differences being undetected.

## Conclusion

The [^18^F]FDOPA-H production method provides a higher activity yield, offering the possibility to scan more patients with one single production. We demonstrated that comparable or slightly higher uptake was present in various organs and tumor lesions in subjects scanned with [^18^F]FDOPA-H compared to [^18^F]FDOPA-L, with considerable overlap being present in the interquartile ranges and relatively small effect sizes. Although the impact on clinical practice may be limited, awareness of this issue may be relevant when performing quantitative evaluations and longitudinal research studies.

## Data Availability

The datasets used and/or analyzed during the current study are available from the corresponding author on reasonable request.

## Abbreviations

AADC: Amino acid decarboxylase
FDOPA: Fluoro-l-3,4-dihydroxyphenyl alanine
LAT: Large amino acid transporter
MAO: Monoamine oxidase
NET: Neuroendocrine tumor
SOR: Striatal-to-occipital ratio
VMAT: Vesicular monoamine transporter
